# Endoscopic Monitoring of Treatment of Indeterminate Intestinal Lesions in a Prospective “Real-Life” Cohort in Eastern India Where Tuberculosis Remains Endemic: Distinguishing Intestinal Tuberculosis From Crohn’s Disease

**DOI:** 10.7759/cureus.75663

**Published:** 2024-12-13

**Authors:** Utpal Jyoti Deka, Rajib Sarkar, Jayanta Kumar Dasgupta, Avik Bhattacharyya, Sukanta Ray, Keya Basu, Gopal K Dhali, Kshaunish Das

**Affiliations:** 1 Gastroenterology, Gauhati Medical College, Guwahati, IND; 2 Gastroenterology, Institute of Postgraduate Medical Education and Research, Kolkata, IND; 3 Radiology, Institute of Postgraduate Medical Education and Research, Kolkata, IND; 4 Surgical Gastroenterology, Institute of Postgraduate Medical Education and Research, Kolkata, IND; 5 Oncopathology, Institute of Post Graduate Medical Education and Research, Kolkata, IND; 6 Gastroenterology, Institute of Post Graduate Medical Education and Research, Kolkata, IND

**Keywords:** anti-inflammatory, anti-tubercular therapy, crohn’s disease, endoscopic follow up, indeterminate lesions, india, intestinal stricture, tuberculosis

## Abstract

Introduction

It is sometimes difficult to differentiate between intestinal tuberculosis (ITB) and Crohn’s disease (CD) in India, as both conditions may mimic each other. The aim was to differentiate ITB from CD in indeterminate intestinal lesions with a therapeutic trial of anti-tubercular therapy (ATT) and follow-up to find out the clinical, endoscopic, radiological, and histological predictors for differentiation between ITB and CD.

Methods

A prospective observational cohort study of patients diagnosed with ITB and CD according to the Asia-Pacific Guidelines in a “real-life” clinical setting was conducted. ITB was diagnosed by Paustian criteria with Logan’s modification. CD was diagnosed according to European Crohn’s and Colitis Organization (ECCO) guidelines. We put the patients with a definite diagnosis of ITB and those with an indeterminate diagnosis on ATT and followed them up clinically, endoscopically, and radiologically. Patients were reassessed clinically, endoscopically, and histologically eight weeks after the start of therapy. They were again evaluated endoscopically and radiologically after completion of six months of ATT. The CD patients continued anti-inflammatory, immunomodulator, biological, and/or steroid treatments.

Results

We conducted this prospective study on consecutive Indian patients who had 21 definite diagnoses of ITB, 26 definite diagnoses of CD, and 42 indeterminate diagnoses. We diagnosed 49 with ITB and 28 (57%) after a therapeutic trial. Ultimately, 40 patients received a CD diagnosis, with 14 (35%) not responding to the ATT therapeutic trial. In patients with ITB, symptomatic improvement after eight weeks of ATT is correlated with endoscopic healing, especially for ulcers but not necessarily for nodularity or strictures. In 50% of these patients, minimal nodularity/pseudopolypii as well as residual scarring was seen on endoscopy even after completion of therapy. Strictures in ITB patients persisted on endoscopy in 40% despite six months of ATT. GI bleeding (64% vs. 10%; p < 0.0001), chronic diarrhea (71% vs. 35%; *P *= 0.02), fistula or sinuses (21% vs. 0%;* p *< 0.01), and multiple site involvement of the intestine (73% vs. 6%; p < 0.0001) were significantly more common in CD than in patients with ITB. Fever (82% vs. 50%; *p *< 0.01) and positive tuberculin tests were more common in ITB patients. PCR positivity and the presence of AFB in smear and culture could be demonstrated in only a small percentage of ITB patients.

Conclusion

Therapeutic trials in indeterminate intestinal lesions can distinguish ITB from CD without significant adverse effects. Strictures in patients with ITB do not resolve in all patients. GI bleeding, chronic diarrhea, fistulas or sinuses, multiple sites of involvement, and fever have the highest accuracy in differentiating ITB from CD.

## Introduction

With the rising burden of inflammatory bowel diseases (IBD), including Crohn’s disease (CD), in areas of the world where tuberculosis is still endemic (e.g., Asia and the Indian subcontinent) [1,2], an increasing dilemma facing clinicians in these regions is the differentiation of intestinal tuberculosis (ITB) vis-à-vis CD [3,4]. This differentiation is clinically important as the two diseases have different long-term prognoses: with a cure achievable in the former, while only remission is achievable in the latter. Furthermore, the use of intense immune suppression to achieve remission in CD can exacerbate and worsen ITB [5]. Conversely, administering anti-tubercular therapy (ATT) to CD patients unnecessarily exposes them to ineffective therapy and delays diagnosis [1,6]. This above clinical conundrum is not only important in tuberculosis-endemic regions of the globe but also in developed areas of the world having a significant immigrant population from these endemic areas [4,5].

The clinical presentations of the two diseases are very similar as they involve similar segments of the small and large intestines, producing similar macroscopic pathologies (viz., ulcerations, strictures, and fistulae) [4]. A plethora of clinical, serological, molecular, endoscopic, radiological, and histological characteristics, either singly or in combination, have been used to differentiate between the two, with limited success [4]. Some of these above investigations are often not in the realm of day-to-day “real-life” clinical practice settings [7].

ITB, like smear-negative pulmonary tuberculosis (PTB), is a pauci-bacillary disease, making the microbiological diagnosis (by smear and/or culture) a difficult and less frequent proposition [8,9]. Despite its inherent disadvantages, empirical ATT has been used in such paucibacillary situations with some degree of success [10,11]. Moreover, expert panel opinions from the Asia-Pacific region have justified the use of an empirical ATT trial for four to eight weeks to differentiate ITB from CD [3]. This implies that there will be significant clinical resolution of symptoms as well as endoscopic and/or radiological healing after the above-mentioned duration of ATT in patients with ITB. Although clinical resolution has been documented in previous reports [12], there has been a relative paucity of data on the endoscopic or radiologic evolution of ITB while a patient is receiving ATT [13]. Most of these studies have looked into the endoscopic or radiological healing of lesions after completion of therapy [14-17], and rarely while therapy is ongoing [15].

With this in mind, we initiated a prospective non-interventional observational cohort study of patients with ITB and CD, the latter diagnosed with or without an empirical ATT-trail of eight weeks, in a “real-life” clinical setting to look into the differentiating clinical features and outcomes of the two. Moreover, the endoscopic evolution of ITB after eight weeks of initiation of ATT was also assessed to identify the relevance of the regional guidelines in the diagnosis of CD.

## Materials and methods

The School of Digestive and Liver Diseases, IPGMER, Kolkata, India, enrolled consecutive patients with symptoms and signs compatible with ITB and/or CD, satisfying the pre-defined inclusion and exclusion criteria, into its outpatient and inpatient gastroenterology and gastrointestinal surgery services from May 2011 to February 2015 (46 months). The hospital is a tertiary-care academic public hospital in the capital of the state of West Bengal in Eastern India. We obtained informed consent from all the patients in their native language.

Patient evaluation

Patients demographics (age, sex), clinical features (pain abdomen, gastrointestinal bleeding, chronic diarrhea, fever, night sweats, weight loss, intestinal obstruction, abdominal lump, fistula, perianal lesions, and extraintestinal manifestations), endoscopic findings (site of involvement, ulcers, nodules, skip lesions, stricture, ileocecal involvement, cobblestone appearance), tuberculin test results, barium or CT enteroclysis findings (bowel thickening, skip lesions, ulceration, segmental involvement, intestinal stricture, type of enhancement, mesenteric changes like fibrofatty proliferation and combs sign, lymph node enlargement, peritoneal thickening or ascites), pathological and microbiological findings were recorded in all patients.

The clinical data of the patients were recorded in Microsoft Excel (Microsoft® Corp., Redmond, WA), and the following investigations were also done at baseline: complete blood counts, erythrocyte sedimentation rate (ESR), liver and renal function tests, stool routine and microscopy, chest X-ray, abdominal X-ray or CT scan (in those suspected of having intestinal obstruction), the Mantoux test (by standard protocol [18]), and trans-abdominal ultrasonography (US). Barium (Microbar™, ESKAY Fine Chemicals, India) contrast studies, i.e., barium-meal follow-through, small bowel enema (enteroclysis), or CT enteroclysis, were done according to standard protocols [19,20].

Ileo-colonoscopy (Olympus GIF-V70, 150 or 170, Fujinon EC-201W2, and Pentax EC-380LKp), enteroscopy (double-balloon endoscopy [DBE], Fujinon Corp., Saitama, Japan), and/or esophago-gastro-duodenoscopy were performed as indicated with documentation of the endoscopic lesions [4,21]. Six to eight biopsies from the endoscopic lesions and adjacent areas were taken for histopathological and microbiological analysis. Presence of caseation, well-formed granuloma (consisting of Langhans’ giant cells with epithelioid cells with a peripheral cuff of lymphocytes), ill-formed granulomas (consisting of small, poorly organized collections of epithelioid cells without the other features of granulomas), ulceration of surface epithelium, appearance of crypts, presence of lymphoid aggregates, and site and type of inflammatory infiltrate were noted. Biopsy specimens were subjected to Ziehl-Neelsen stain for detection of AFB. Biopsy specimens were also sent for TB-PCR for amplification of the insertion element IS6110. Oligonucleotide primers for the detection of Mycobacterium tuberculosis were selected to amplify a 123-base pair (bp) fragment of the 5/ portion of IS6110. The sequences of the primers used (5/ to 3/) were CCTGCGAGCGTAGGCGTCGG and CTCGTCCAGCGCCGCTTCGG. BACTEC/MGIT (Mycobacteria Growth Indicator Tube) cultures for M. tuberculosis were done from biopsy specimens according to established laboratory procedures.

Inclusion criteria comprise consecutive patients presenting with symptoms and signs consistent with ITB and/or CD, such as chronic abdominal pain, partial obstruction, diarrhea (>4 weeks), GI bleeding, fistulae, or altered bowel habits. Exclusion criteria include HIV-positive status, pregnancy, gastrointestinal malignancy, significant comorbidities (e.g., coronary artery disease, chronic kidney disease, cirrhosis, advanced COPD), or prior anti-tubercular therapy for TB.

Diagnosis

The diagnosis of ITB was made on the basis of Paustian criteria with Logan’s modification. There were three groups of patients: group I (definite ITB) who had characteristic clinical and endoscopic and/or radiological features [4,21] with positive microbiology (presence of acid-fast Bacilli on the smear/culture or positive finding of the insertion element IS6110 by polymerase chain reaction) or characteristic histological features (either the presence of caseating granulomas or non-caseating well-formed granulomas composed of epitheloid cells with a peripheral cuff of lymphocytes having Langhans giant cell); group II (indeterminate) who had the same clinical and endoscopic and/or radiological features as group I patients but negative microbiology and histology; and, group III (definite CD) who satisfied the European Crohn’s and Colitis Organization (ECCO) guidelines, i.e., a combination of clinical, endoscopic and histological features of Crohn’s disease. Group II patients were diagnosed as having ITB if they were responders (cf. below) to eight weeks of a therapeutic trial of ATT, while non-responders (cf. below) were re-evaluated [3].

Treatment

Patients diagnosed with CD (group III) received a tapering dose of oral steroids for three months, as well as 5-ASA with/without azathioprine at a dose of 1.25 mg/kg or biologicals to maintain remission. Groups I and II patients were started on a short course of chemotherapy (six months of ATT) [12,15]. This consisted of a two-month intensive phase of isoniazid (5 mg/kg), rifampicin (10 mg/kg body weight), ethambutol (15 mg/kg), and pyrazinamide (25 mg/kg). After this phase, both groups I and II patients were again reassessed clinically and endoscopically. Group II patients who had clinical and endoscopic responses, and all group I patients received the continuation phase of four months of ATT, consisting of isoniazid (5 mg/kg) and rifampicin (10 mg/kg body weight). All patients on ATT also received daily pyridoxine supplementation. Patients having adverse drug reactions like drug-induced liver injury, neurotoxicity, or nephrotoxicity underwent drug modifications as per standard recommendations. In only one patient with markedly deranged liver enzymes at baseline, second-line anti-tubercular therapy (ofloxacin, ethambutol, and streptomycin) was started at baseline.

Group II patients not responding to the intensive phase of ATT were re-evaluated and diagnosed as CD. ATT was stopped in them, and they were started on steroids or biologicals and 5-ASA with or without concomitant azathioprine and followed up.

Follow-up in groups I and II patients

A detailed evaluation of clinical symptoms and physical examination was done during follow-up at one month, two months, four months, and six months after starting ATT, and then at three- to six-month intervals after the completion of ATT for at least five years till December 2020. Clinical response, assessed at the end of six to eight weeks of ATT, was categorized as responders or non-responders. Patients who became asymptomatic or who had significant symptomatic improvement (as assessed by the treating physician) were designated as responders. In contrast, those in whom symptoms remained the same or further deteriorated were designated as non-responders. Endoscopic reassessment was also done at this time (after six to eight weeks of ATT) and again at six months (after completion of the continuation phase of ATT). During follow-up endoscopy, the degree of healing of baseline lesions was assessed, and a biopsy was taken. Repeat barium radiography was done in those with a baseline abnormal study at six months after completion of ATT.

Group III patients and CD diagnosed after nonresponse to ATT from group II were also followed up at the interval of one to two months for three years, thereafter three to six monthly for a total of at least five years.

Statistical analysis

Categorical variables were presented as percentages, and continuous data as median and range. For univariate analysis, Pearson’s chi-squared test or Fisher’s exact test was used for categorical variables, while Mann-Whitney U and Kruskal-Wallis H were used for continuous variables. All statistical analysis was done by SPSS software for Windows (version 19.0, Chicago, IL).

The Institute Ethical Committee (IEC) provided ethical clearance via Memo No. Inst/IEC/1186, dated May 5, 2011.

## Results

**Figure 1:**
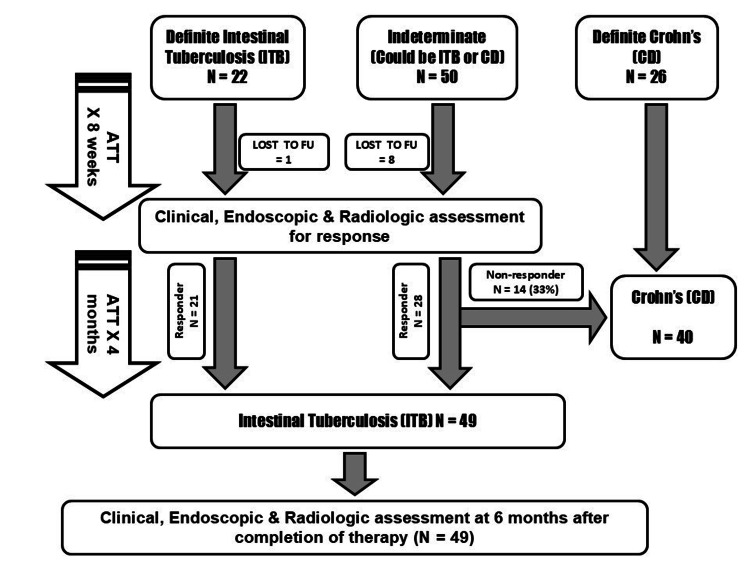
Study flowchart (STROBE diagram).

Figure 1 depicts the patient flowchart. We enrolled 98 patients but lost 9 to follow-up. Forty patients were ultimately diagnosed to have CD, 14 (35%) after non-response to the intensive phase of ATT. Forty-nine patients were finally diagnosed to have ITB, 28 (57%) after a therapeutic trial of ATT.

Table 1 shows the baseline demography, clinical features, and investigations in the three groups of patients. There was no difference in the age and sex ratios in the three groups, with patients in group III, i.e., those diagnosed to have definite CD, having symptoms for the longest time before diagnosis. Group II patients, in whom ITB or CD could not be established without a therapeutic trial, presented with partial intestinal obstruction less frequently and had clinically significant ascites more frequently than the other two groups. These group II patients also had an intermediate frequency of Mantoux positivity and had an abnormality on barium radiography the least frequently. They involved the colon and ileum in a distribution that was similar to group I patients on the right-colonic side and to group III patients on the left-colonic side. There was no difference in the type of lesions on endoscopy or barium radiography (viz., ulcers, nodules, strictures, etc.) between the three groups.

**Table 1:** Baseline demography, clinical features, and investigations. ^1^Cecum, ascending colon and hepatic flexure. ^2^Splenic flexure, descending and sigmoid colon.

	Group I definite ITB (N=21)	Group II indeterminate (N=42)	Group III definite Crohn’s (N=26)	P-value
Age (median, range), years	27.5 (14–45)	28.0 (14–59)	31.0 (24–60)	NS
Males (%)	13(62)	21(50)	17(65)	NS
Symptom-onset to diagnosis duration (median, range), months	8.5 (3–36)	10.0 (1–72)	42.0 (2–120)	<0.0001
Presenting symptoms and signs				
Abdominal pain (%)	18(86)	36(86)	24(92)	NS
Partial intestinal obstruction (%)	9(43)	9(21)	13(50)	0.04
GI bleed (%)	2(10)	12(29)	11(42)	0.05
Palpable abdominal mass (%)	5(24)	2(5)	4(15)	NS
Ascites (%)	0	9(21)	2(8)	0.04
Pyrexia (%)	17(81)	31(74)	8(31)	<0.0001
Unintentional weight loss (%)	18(86)	29(69)	21(81)	NS
Laboratory investigations				
Hemoglobin (median, range), g/dL	10.1 (4.9–14.6)	9.4 (3.4–14.0)	9.6 (6.0–14.0)	NS
ESR (1^st^ hour) (median, range), mm	54 (13–80)	48 (10–130)	50 (11–110)	NS
Platelets (median, range), × 10^5^/mm^3^	2.5 (1.5–6.4)	2.5 (1.3–4.3)	3.0 (1.9–4.2)	<0.01
TST-positive [5TU] (%)	16(76)	19(45)	5(19)	0.001
Radiology				
Abnormal chest X-ray (%)	3(14)	2(5)	1(4)	NS
Ascites on ultrasound (%)	4(19)	9(21)	5(19)	NS
Lymphadenopathy on ultrasound (%)	5(24)	5(12)	1(4)	NS
Thickened bowel wall in ultrasound (%)	5(24)	5(12)	3(12)	NS
Abnormal barium radiography (%)	14(68)	21(50)	18(69)	0.04
Stricture	13(63)	13(31)	12(46)	0.06
Ulcer	5(24)	3(7)	7(27)	0.08
Deformed cecum	8(38)	10(24)	4(15)	NS
Ileo-colonoscopy/enteroscopy				
Ileum normal (%)	6(28)	13(31)	3(12)	NS
IC-valve normal (%)	7(33)	16(38)	19(73)	0.005
Right-colon involved^1 ^(%)	15(71)	29(69)	10(39)	0.05
Left-colon involved^2^ (%)	1(5)	11(26)	8(31)	0.08
Histopathology				
Granuloma present (%)	20(95)	13(31)	6(23)	<0.0001
Well-formed granuloma (N=68) (%)	20(95)	2(5)	0	<0.0001
Caseation (%)	3(14)	0	0	0.006
TB PCR positive (%)	5(24)	0	0	0.004
AFB positivity (%)	2(10)	0	0	0.007
AFB culture (%)	4(19)	0	0	0.004

Seventy-two patients (groups I and II) started on ATT; nine were lost to follow-up despite repeated reminders. After eight weeks of therapy, 14 (22%) who had no symptomatic improvement and/or had worsening symptoms were categorized as non-responders and were later diagnosed with CD. The remainder were diagnosed to have ITB and were considered responders, out of which 31 (49%) became completely asymptomatic and 18 (29%) had significant symptomatic improvement. Table 2 compares the responders with the non-responders. Non-responders had a longer duration of symptoms, presented more frequently with overt/occult GI bleeding and diarrhea, and less frequently had pyrexia or weight loss. Fistula and/or sinuses were exclusively seen in them. They were more hypoalbuminemic and more frequently had pedal edema. There was no difference in the frequency of abnormal ultrasound or barium radiography, although they were less frequently Mantoux positive. On endoscopy, although the types of lesions were not different between the two, there was a significant difference in the distribution of the lesions. Despite no difference in the frequency of granulomas overall, well-formed granulomas were characteristically absent in the non-responders, and cryptitis was more frequent in them. Endoscopically, responders showed healing of ulcers and nodules at the end of eight weeks of ATT. However, persistent strictures were present with similar frequency in both groups.

**Table 2:** Comparison of responders with non-responders to eight weeks of therapeutic trial of anti-tubercular therapy.

	Yes (N = 49)	No (N = 14)	P-value
Age (median, range), years	27.5 (14–58)	30.0 (20–59)	NS
Males (%)	26(53)	7(50)	NS
Symptom-onset to diagnosis duration (median, range), months	8.0 (1–36)	12.0 (2–24)	0.03
Presenting symptoms and signs			
Abdominal pain (%)	44(90)	10(71)	NS
Partial intestinal obstruction (%)	15(31)	4(29)	NS
GI bleed (%)	5(10)	9(64)	<0.0001
Diarrhea (%)	17(35)	10(71)	0.02
Fistula and/or sinus (%)	0	3(21)	<0.01
Ascites (%)	5(10)	4(29)	NS
Pedal edema (%)	5(10)	5(36)	0.03
Pyrexia (%)	41(84)	7(50)	<0.01
Weight loss (%)	40(82)	8(57)	0.06
Laboratory investigations			
Hemoglobin (median, range), g/dL	10.0 (3.4–14.6)	8.8 (4.6–11.2)	NS
Platelets (median, range), × 10^5^/mm^3^	2.5 (1.3–6.4)	2.8 (1.6–4.0)	NS
Albumin (median, range), g/dL	3.2 (1.2–5.2)	2.5 (1.6–3.4)	0.02
TST-positive [5TU], %	35(71)	2(14)	<0.0001
Radiology			
Abnormal chest X-ray, %	5(10)	0	NS
Abnormal ultrasound, %	19(39)	6(43)	NS
Abnormal barium radiography, %	26(53)	7(50)	NS
Ileo-colonoscopy/enteroscopy			
Ileum normal; N = 59, %	15(31)	3(21)	NS
Right-colon involved^1^, %	35(71)	9(64)	NS
Ileocecal involvement, %	37(76)	6(43)	0.02
Transverse-colon involved, %	2(4)	8(54)	<0.0001
Left-colon involved^2^, %	1(2)	11(79)	<0.0001
Ulcers, %	42(86)	14(100)	NS
Nodules, %	33(67)	7(53)	NS
Strictures, %	12(25)	4(29)	NS
Histopathology			
Granuloma present, %	29(59)	5(36)	NS
Well-formed granuloma (N = 34), %	23(68)	0	<0.0001
Caseation, %	3(6)	0	NS
AFB Positive, %	2(4)	0	NS
TB PCR, %	5(10)	0	NS
TB culture, %	4(8)	0	NS
Cryptitis, %	6(12)	5(36)	0.04
Endoscopic response at 2 months			
Ulcers, complete disappearance, %	29(69)	0	<0.0001
Nodules healed (N = 43), %	32(97)	0	<0.0001
Persistent stricture, %	9(19)	4(29)	NS
Outcome			
Surgery, %	2(4)	3(21)	0.03

All patients underwent colonoscopy with ileal intubation at baseline; ileal intubation was unsuccessful in 5. Eight patients underwent enteroscopy (retrograde in 4 and bi-directional in the remainder). Repeat endoscopies were done in all patients at eight weeks and at the end of six months of ATT. Barium studies were available in all patients at the end of 6 months of ATT.

Table 3 shows the endoscopic and radiological evolution of tubercular lesions during therapy. On endoscopy, 42 (86%) patients had ulcers at baseline, which resolved completely in 29 (69%) by two months, while in the remaining 13 with partial healing, complete resolution was observed in all in whom a repeat study was available at six months.

**Table 3:** Endoscopic and radiological evolution of tubercular lesions during therapy. *Visible but healing ulcers and nodules. ^#^Healed nodules with pseudopolyps.

	Baseline (N=49)	At 8 weeks (N=49)	At 6 months (N=41)
Ileo-colonoscopy or enteroscopy			
Ulcers (%)	42(86)	11(26)^*^	0
Nodules (%)	33(67)	16(49)^*^	11(33)^#^
Polypoidal lesions (%)	2(4)	0	0
Strictures (%)	12(25)	6(12)	6(12)
Erythema (%)	49(100)	4(8)	0
Erythema and exudates (%)	26(53)	0	0
Barium radiography			
Normal, N (%)	23 (47)		
Abnormal, N (%)	26 (53)		13 (50)
Ulcers (%)	14(29)		0
Strictures (%)	18(37)		6(12)
Deformed cecum (%)	14(29)		7(14)

**Figure 2:**
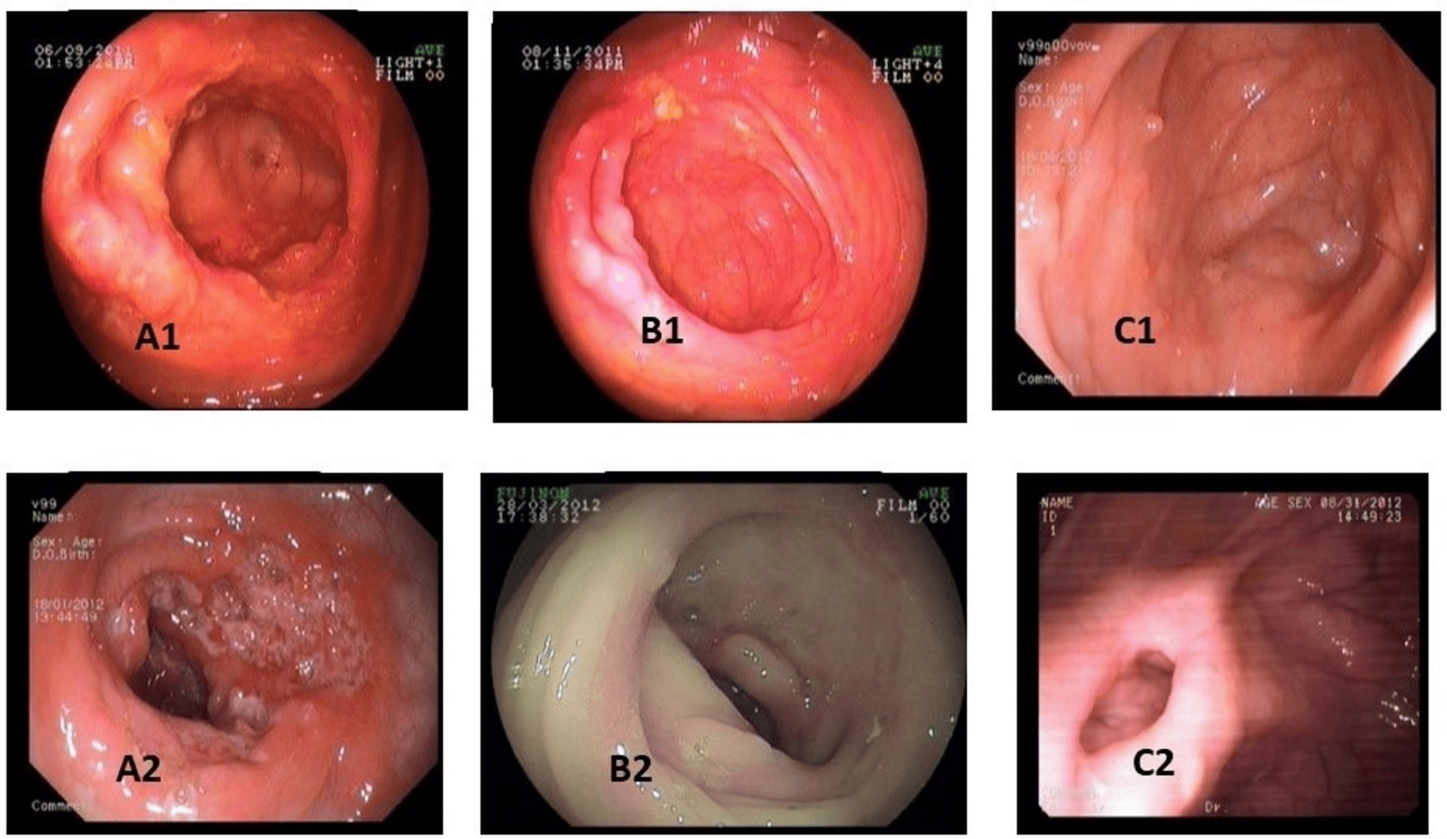
Endoscopic healing of ileocaecal tubercular lesions with short course anti-tubercular therapy (ATT). A1A2: before ATT; B1B2: at two months of ATT; C1C2: after six months of ATT.

**Figure 3:**
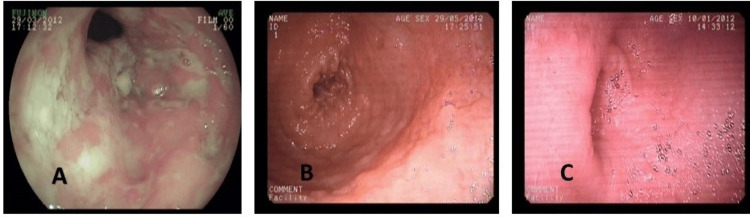
Endoscopic healing of ileal tuberculosis with short course anti-tubercular therapy (ATT). (A) Before ATT; (B) at two months of ATT; (C) after six months of ATT.

**Figure 4:**
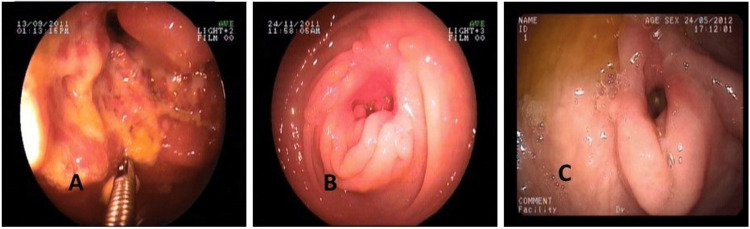
Endoscopic evolution of tubercular intestinal stricture with anti-tubercular therapy (ATT). (A) Before ATT; (B) at two months of ATT; (C) at six months of ATT.

Figures 2-3 show the endoscopic healing of ileocecal and only ileal ITB lesions with a short course of ATT at two months and six months, respectively. Only one patient with an ulcer at baseline evolved into a stricture after six months of ATT despite endoscopic healing of the ulcers. Thus, ulcers healed in all patients after completion of ATT. Twelve ITB patients had strictures at baseline on endoscopy (two colonic, six ileocecal, four ileal). After two months of ATT, complete resolution of strictures was seen in six (50%). In the remaining six patients, after six months of ATT, strictures persisted in five (one colonic, three ileocecal, and one ileal). Thus, 58% had endoscopic resolution of strictures after six months of ATT. Overall, six patients had residual strictures on endoscopy after completion of ATT. Figure 4 shows the endoscopic evolution of intestinal tuberculosis stricture during follow-up at two months and at the end of therapy. Thirty-three ITB patients had nodules on endoscopy at baseline (21 with ulcers alone, 8 with stricture, and 4 with only nodules). Seven patients (21%) had complete resolution of nodules endoscopically after two months of ATT. After six months of ATT, of the 26 patients with residual nodularity at two months, 13 (50%) had complete resolution, while healed pseudo-polypi were seen in the other 13 patients (five with associated persistent stricture). Overall, 20 patients (61%) had complete resolution of nodules after completion of ATT and healed pseudo-polypii persisting in the remainder. On endoscopy, 26 (53%) ITB patients had ileo-cecal valve (ICV) involvement at baseline (23 with ulceration, 22 with nodularity, and 6 with stricture). After six months of ATT, healed but deformed IC-valve was seen in 11 (4 with associated persistent stricture and 9 with pseudo-polypi), while complete resolution was seen in the remaining 15 (58%) patients.

**Figure 5:**
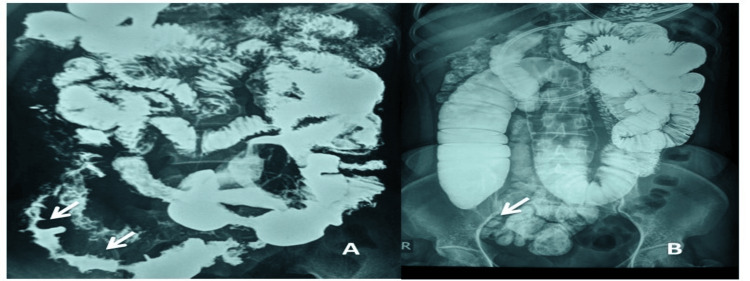
(A) Stricture with ulceration (white arrow); (B) persistence of stricture with healing of ulcers (white arrow) on barium studies.

Of the 49 ITB patients with barium radiography at baseline, lesions were detected in 26 of them. Twenty-three of them had ileocecal disease, with only three having isolated small bowel involvement. A repeat barium study was done after six months of ATT in 26 of these patients with baseline abnormalities. Of the 18 patients with strictures in barium at baseline, 5 (28%) had persistent strictures after 6 months of therapy. Overall, six patients had residual strictures after completion of ATT. All of them exhibited the same symptoms during the endoscopy. Five of these six patients also had an associated deformed cecum on barium radiography. Fourteen patients had cecal involvement at baseline, which resolved completely in seven (50%) patients, while in the other seven healed but radiologically deformed cecum was found at the end of six months. Figure 5 shows the evolution of stricture with ulceration and the persistence of stricture with the healing of ulcers in barium studies.

The six ITB patients with persistent strictures, despite completion of ATT, did continue to have episodes of symptomatic partial intestinal obstruction, though in lesser frequency and intensity, for the post-therapy six years of follow-up. Compared to the remaining patients with ITB, these six patients were more likely to have palpable abdominal mass (50% vs. 8%, respectively; p < 0.01) and symptoms of partial intestinal obstruction (100% vs. 24%, respectively; p < 0.0001) at presentation. At the baseline evaluation, they were also more likely to have strictures observed on endoscopy (83% vs. 24%, respectively; p < 0.0001) and more likely to have caseating granulomas on histology (33% vs. 3%, respectively; p < 0.01). No other differences in age, sex, symptom duration, laboratory parameters, ultrasound, barium radiography, and endoscopic distribution of lesions were noted at baseline between the two groups. During over six years of follow-up, one remained asymptomatic; three had mild intermittent intestinal colic that responded to anti-spasmodic medication; two underwent surgical resection after eight months to two years of completion of ATT. Histopathological examination of the surgical specimen showed no granuloma or any features of active inflammation. Follow-up endoscopic biopsies in the remainder showed no evidence of active inflammation or granulomas.

**Figure 6:**
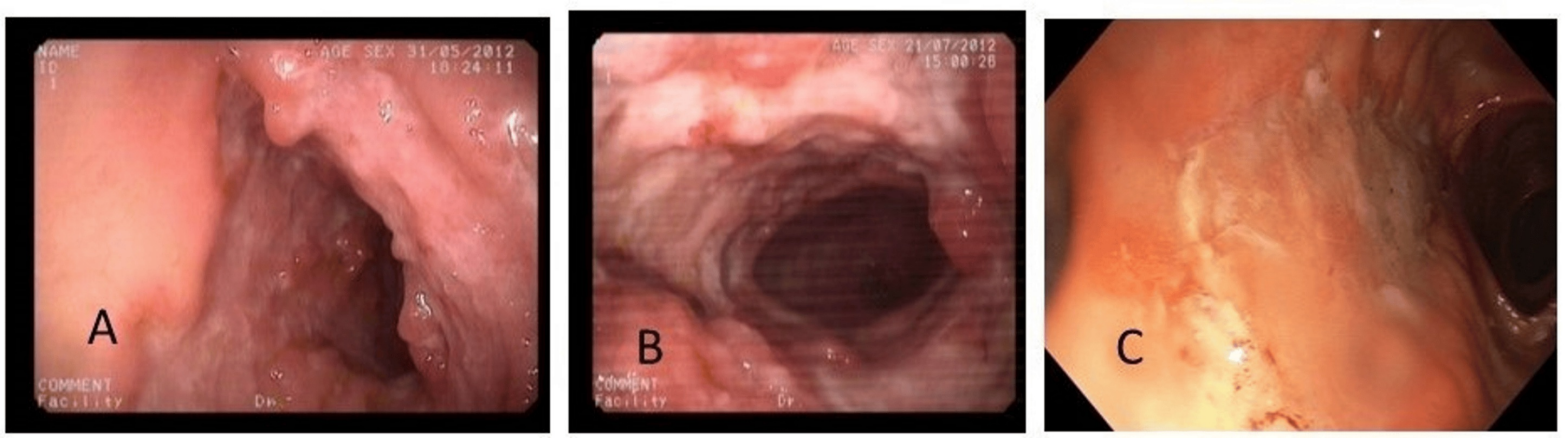
Endoscopic features of an indeterminate patient who was subsequently diagnosed as Crohn’s with (A) baseline lesion before anti-tubercular (ATT); (B) after eight weeks of ATT, aggravation of lesions; (C) at two months after steroid and mesalamine, significant healing.

Group III patients (CD) and CD diagnosed after non-response to ATT from group II were also followed up clinically and endoscopically. Follow-up endoscopy was done in 40 CD patients at eight weeks after starting therapy (corticosteroid and 5ASA with or without azathioprine or biologicals), and all CD patients showed signs of healing of lesions. Figure 6 shows the endoscopic features of an indeterminate patient who was subsequently diagnosed as CD with aggravation of lesions with eight weeks of ATT and significant healing with steroid and anti-inflammatory medicines.

Table 4 shows the difference between ITB and CD patients. CD patients were diagnosed late and presented more frequently with diarrhea (71% vs. 35%; P = 0.02), gastrointestinal bleeding (64% vs. 10%; p < 0.0001), fistulae/sinuses (21% vs. 0%; p < 0.01), and multiple intestinal site involvement (73% vs. 6%; p < 0.0001) than patients with ITB. On colonoscopy, right colonic involvement was more frequent in ITB patients, while left colonic involvement was more common in CD. Caseation and demonstration of AFB in smear were found in only 6% and 4% of ITB patients, respectively. AFB culture positivity and TB PCR positivity were also low, only in 8% and 10% of intestinal biopsies of ITB patients. So, demonstration of AFB in smear or culture and positivity of TB PCR were found in small numbers of ITB patients in our country.

**Table 4:** Differences between intestinal tuberculosis and Crohn’s disease patients at baseline. ^1^Cecum, ascending colon and hepatic flexure. ^2^Splenic flexure, descending and sigmoid colon.

	ITB (N=49)	Crohn’s (N=40)	P-value
Age (median, range), years	28 (14–58)	30 (20–60)	NS
Males (%)	26(53)	25(62)	NS
Symptom-onset to diagnosis duration (median, range), months	8 (1–36)	24 (2–120)	<0.0001
Presenting symptoms and signs			
Abdominal pain (%)	44(90)	34(85)	NS
Partial intestinal obstruction (%)	15(31)	16(40)	NS
Diarrhea (%)	17(35)	23(58)	0.04
GI bleed (%)	5(10)	20(50)	<0.0001
Palpable abdominal mass (%)	7(14)	4(10)	NS
Fistula or sinus (%)	0	6(15)	<0.01
Pedal edema (%)	5(10)	12(30)	0.02
Pyrexia (%)	41(84)	15(38)	<0.0001
Weight loss (%)	40(82)	28(70)	NS
GI-tract involvement			0.07
Upper GI (%)	4(8)	0	
Small-bowel alone (%)	4(8)	9(23)	
Ileo-colonic (%)	34(69)	27(68)	
Colon alone (%)	7(14)	4(10)	
Colonoscopic distribution of lesions			
Ileum (%)	34(69)	33(83)	NS
IC-valve spared (%)	17(35)	25(62)	0.01
Cecum (%)	32(65)	18(45)	NS
IC-valve + cecum (%)	37(76)	17(42)	<0.01
Right-colon involved^1^ (%)	35(71)	19(48)	0.03
Transverse Colon involved (%)	2(4)	16(40)	<0.0001
Left-colon involved^2^ (%)	1(2)	19(48)	<0.0001
Stricture at endoscopy (%)	12(25)	14(35)	NS
Multiple site involvement (%)	3(6)	29(73)	<0.0001
Barium radiography			
Normal (%)	23(47)	16(40)	NS
Ulcers (%)	14(29)	8(20)	NS
Strictures % (number)	18(37)	18(45)	NS
Deformed cecum % (number)	23(47)	6(15)	0.01

Of the 40 patients diagnosed with CD in our cohort, 14 (35%) were diagnosed after an ATT trial. There were no differences in the two groups of CD patients diagnosed with or without ATT-trail with respect to baseline demography, symptom-diagnosis interval, presenting signs and symptoms, laboratory parameters, ultrasound, and barium radiography. The only difference was that, on colonoscopy, those who were diagnosed after an ATT trial were less likely to have a normal ileocecal valve (38% vs. 75%, respectively; p = 0.03) and more likely to have simultaneous ascending colon and left colon involvement (57% vs. 15%, respectively; p < 0.01). In these patients with six years of follow-up, there was no difference in the need for surgery or immune modulators between these two groups. Three CD patients with stricture underwent surgery during this follow-up period. All patients were continuing anti-inflammatory (5ASA) with or without immunomodulators (azathioprine), biologicals, and sometimes steroids with flares.

## Discussion

ITB and CD are both chronic granulomatous diseases with various similarities in their clinical, radiological, endoscopic, and histological features; thus, the differential diagnosis of these two conditions remains a major problem for clinicians. In India, as more and more cases of CD are being now recognized, the definite diagnosis of CD or intestinal tuberculosis becomes increasingly important [9]. Over the last few years, there have been some published articles from India giving importance to intestinal tuberculosis and trying to differentiate intestinal tuberculosis from Crohn’s disease [22,23]. Differentiation of intestinal tuberculosis and CD remains a big problem, especially in some patients presenting with indeterminate intestinal lesions, and there is still no simple test for differentiating the two granulomatous diseases.

It is seen from our study that among 49 ITB patients, 28 (57%) were diagnosed after a therapeutic trial of ATT, and 35% of Crohn’s were diagnosed after seeing nonresponse to ATT. There is no difference in age and sex ratio among the patients with ITB and CD. CD patients have symptoms for the longest time before diagnosis. This is due to the diagnostic delay and the remitting-relapsing course of Crohn’s patients. The reason for the high number of ITB patients (57%) diagnosed after ATT is the referral of the patients having a diagnostic dilemma. Clinically, CD patients (diagnosed after nonresponse to ATT) had a longer duration of symptoms, presented more frequently with overt/occult GI bleed, diarrhea, fistula, or sinuses, and less frequently had pyrexia or weight loss than ITB patients, which was as shown in some previous studies [22,23].

The indeterminate group of patients had a less frequent abnormality in barium radiography than definite ITB or Crohn’s disease in our study. There are no differences in types of lesions (ulcers, nodules, strictures, etc.) among the patients with definite ITB or CD disease and the indeterminate group of patients. Right-sided colonic involvement with or without ileal involvement is more common in ITB than with CD. Left colonic involvement is more with CD. Multiple site involvement with normal intervening mucosa of the intestine is also more significantly found in CD.

Transverse ulcers, nodularity, and hypertrophic mass-like lesions in ITB and deep linear fissuring ulcers with a cobblestone appearance in CD are classically described in the endoscopy of these two conditions. However, in reality, many patients either lack these classic findings or exhibit overlapping findings, which makes it challenging to distinguish between ITB and CD. In clinical practice, nonspecific ulcers and nodules on the ileocecal valve and cecum without typical features of CD or ITB are often seen [13]. In our study, also, ulcers, nodules, and strictures did not differentiate ITB from CD in most of the cases. There was a significant difference in the distribution of the lesions in endoscopy, and left-sided colonic involvement was found to be more in CD than ITB, as previously described in some studies [23,24]. Large, well-formed granulomas, caseation often with AFB, confluent granulomas, and large numbers of granulomas are considered to be diagnostic of intestinal TB, but these histological changes are often found mostly in surgically resected specimens. Caseation and AFB were found in only 6% and 4% of ITB patients, respectively. The histological differentiation of intestinal TB and CD is most commonly tried on endoscopic mucosal biopsies. However, only a small number of patients with mucosal biopsies exhibit these histologic characteristics of ITB, which help differentiate it from CD. In intestinal TB, there was caseation in 18% and the confluence of granulomas in 15.4% in one study [25], and granulomas >5/section and granuloma size > 200 µm in 22% and 27%, respectively, in ITB patients in another study [23]. In our study, caseation and TB PCR positivity were seen only in 6% and 10% of ITB patients, which are not good sensitive tests for diagnosis of ITB. AFB was positive only in two (4%) patients of ITB in our study, which was also previously reported in a small percentage of ITB patients from our country [22].

If the clinical and endoscopic features are suggestive of tuberculosis and multiple target biopsies do not show evidence of any other disease, then a therapeutic trial of anti-tuberculosis treatment can be given to these patients following up [13]. An empirical ATT trial for four to eight weeks for differentiating ITB from CD can be considered in the Asia-Pacific region in indeterminate intestinal lesions when the diagnosis is not conclusive [3]. However, the response should be assessed with close and frequent monitoring of the patient after putting on empirical ATT. Only clinical monitoring may be misleading, as some CD patients may show little symptomatic improvement with ATT. So, endoscopic documentation of healing at eight weeks may be considered to establish an appropriate diagnosis and to continue therapy.

In patients with ITB, symptomatic improvement after eight weeks of ATT is correlated with endoscopic healing, especially for ulcers but not necessarily for nodularity or strictures. In up to half of these patients, endoscopically minimal nodularity/pseudo-polypi, as well as residual scarring on endoscopy and/or barium radiography, can be seen even after the completion of six months of therapy. Importantly, in patients of ITB who have strictures at baseline, they persist endoscopically in up to 40% and on barium radiography in up to 28%, even after the completion of six months of ATT.

Although a number of researchers have used a variety of molecular tests [7,24] or computed tomography [25], or a combination of clinical and endoscopic and/or histologic scores [26-28] to achieve point-of-care distinction between ITB and Crohn’s disease, the majority of them are retrospective, and none have been externally validated. The role of these new molecular tests is also not known in patients presenting with indeterminate intestinal lesions. In this regard, our study prospectively validates a diagnostic algorithm that serves as a consensus guideline in tuberculosis-endemic regions of Asia-Pacific [12]. Previously another study had tried to validate this diagnostic algorithm in a retrospective-prospective manner [29].

Significant healing of ulcers with therapy can be documented on repeat endoscopy, which is important in indeterminate lesions. However, in all 14 patients who subsequently turned out to be CD, there were no signs of healing of ulcers, nodules, and strictures endoscopically at eight weeks. However, there was a mild clinical improvement with ATT in four patients. So, endoscopic documentation of healing at eight weeks may be very important regarding the continuation of ATT after two months of therapy in suspicious cases of intestinal tuberculosis. All CD patients showed clinical and endoscopic responses to steroids, anti-inflammatories, biologicals, and/or immunosuppressants on follow-up. CD patients were diagnosed late and presented more frequently with diarrhea, gastrointestinal bleeding, fistulae/sinuses, multi-segment intestinal involvement, and pedal edema than patients with ITB. On colonoscopy, right colonic involvement was more frequent in ITB patients, while left colonic involvement was more common in CD.

The strength of the study is that the post-treatment follow-up is at least five years after completion of ATT, which is important in differentiating it from Crohn's disease, which is a remitting and relapsing disease. Though there are some studies that are trying to differentiate ITB from CD, there is a paucity of data on the indeterminate intestinal lesions to find out differentiation during treatment with endoscopic follow-up. Endoscopic follow-ups with the treatment of indeterminate lesions are also compared with endoscopic follow-ups of diagnosed ITB and CD.

Our study has some limitations. Besides being conducted in a tertiary-care center with its inherent bias of attracting the most severe and indeterminate disease phenotypes, the number of patients included was small. Giving an empirical ATT in an indeterminate group to differentiate ITB from CD may not always be acceptable at first presentation.

## Conclusions

In conclusion, on the basis of clinical, radiological, endoscopic, and histological findings, it is sometimes difficult to differentiate ITB from CD in some indeterminate intestinal lesions in India due to the similarities in the presentation of these two diseases. In patients with ITB, symptomatic improvement after eight weeks of ATT correlated with endoscopic healing, especially of ulcers but not necessarily of nodularity or strictures. It is important to document the healing of intestinal lesions endoscopically along with clinical response after starting ATT at two and six months in our country. Stricture resolution occurs in a little higher than half of the stricture with six months of ATT. The CD presents more frequently with diarrhea, GI bleeding, fistula, and multiple site involvements than ITB.
